# The chronobiology of migraine: a systematic review

**DOI:** 10.1186/s10194-021-01276-w

**Published:** 2021-07-19

**Authors:** Amanda Holmen Poulsen, Samaira Younis, Janu Thuraiaiyah, Messoud Ashina

**Affiliations:** Danish Headache Center, Department of Neurology, Rigshospitalet Glostrup, Faculty of Health and Medical Sciences, University of Copenhagen, Valdemar Hansen Vej 5, DK-2600 Glostrup, Denmark

**Keywords:** Migraine, chronobiology, periodicity, circadian, seasonal, weekly

## Abstract

**Background:**

The paroxysmal nature of migraine is a hallmark of the disease. Some patients report increased attack frequency at certain seasons or towards the end of the week, while others experience diurnal variations of migraine attack onset. This systematic review investigates the chronobiology of migraine and its relation to the periodicity of attacks in existing literature to further understand the oscillating nature of migraine.

**Main body:**

PubMed and Embase were systematically searched and screened for eligible articles with outcome measures relating to a circadian, weekly or seasonal distribution of migraine attacks. We found that the majority of studies reported morning hours (6 am–12 pm) as the peak time of onset for migraine attacks. More studies reported Saturday as weekly peak day of attack. There was no clear seasonal variation of migraine due to methodological differences (primarily related to location), however four out of five studies conducted in Norway reported the same yearly peak time indicating a possible seasonal periodicity phenomenon of migraine.

**Conclusions:**

The findings of the current review suggest a possible role of chronobiologic rhythms to the periodicity of migraine attacks. Future studies are, however, still needed to provide more knowledge of the oscillating nature of migraine.

## Background

The paroxysmal nature of migraine is a hallmark of the disease [1]. The frequency of attacks and pain-free intervals vary from patient to patient [2]. Some patients may experience increased frequency of attacks at certain times of the year [3] or towards the end of the week [4], while others report diurnal variations of migraine attack onset [5]. The mechanisms underlying the oscillating nature of migraine are unknown.

Chronobiology is the study of biological rhythms present in most life forms that inhabit the surface of the planet. The best known is the circadian ~24-hour rhythms of behavior, metabolism and physiology. There are also biological rhythms that correspond to phases of the moon or the season, while the ultradian rhythms have periods varying from several hours to minutes. Circadian rhythms have the most pervasive influence on the many functions in the human body such as blood pressure [6], sleep-wake cycles, body temperature and hormone production [7]. Circadian dysfunction is common in shift workers (~ 21 % of workers in the European Union [8]) and has been linked to a large number of health problems either as a cause or an effect [9–11].

In migraine, it is well established that the menstrual cycle influences the periodicity of migraine attacks for some women [12]. Migraine patients may also experience headaches awakening from sleep possibly triggered by sleep disturbances [13]. Moreover, the prevalence of migraine increases with age and peaks at 35–39 years of age, followed by a decline, showing that migraine prevalence changes according to lifespan [14]. The question is whether chronobiology, involving circadian or infradian (> 24 h) biological rhythms, influences the periodicity of migraine attacks. This aspect of periodicity in migraine has been investigated based on data collected from headache diaries, questionnaires and emergency department visits.

Here, we conducted a systematic review of the literature to identify studies that investigated the temporal distribution of migraine attacks within a circadian, weekly and seasonal time frame.

## Methods

### Study identification

This review has been conducted in accordance with the Preferred Reporting Items for Systematic Reviews and Meta-analyses (PRISMA) reporting guidelines [15]. We searched PubMed and Embase to identify all possibly relevant articles. Following search string was used: *Migraine AND (periodicity OR circadian OR circannual OR life span OR chronobiology OR chronology OR rhythm OR cycle OR fluctuation OR rhythmicity OR season OR nocturnal OR equinox OR solstice OR diurnal).* The search was performed August 24th, 2020.

### Study selection

Investigators AHP and JT independently screened all articles by title and abstract. Possible eligible articles were retrieved for full-text screening according to the pre-defined selection criteria (Table 1). Any discrepancy between the two investigators was determined by discussion. Further disagreement between the two investigators was determined by consulting a senior investigator (SY). Subsequently, a manual reference screening of the included studies and other relevant primary articles was performed to find possible eligible studies missed by the search string.

**Table 1:** Selection criteria

Inclusion criteria	Exclusion criteria
English studies	Reviews, meta-analyses, case reports and/or conference proceedings, conference abstracts
Must be diagnoses by a physician with migraine (1.1) or migraine with aura (1.2) according to IHS Classification ICHD-3 (or earlier editions)	Animal studies
Study outcomes must live up to 1 of 2: • Hourly (or in intervals) distribution of attack onset • Weekly and/or seasonal/yearly distribution of migraine attacks or distribution of emergency-department visits	Intervention studies

### Data extraction and analysis

Data were extracted using a pre-defined form. For each included study, data on the following parameters were extracted: study site (country), study type (questionnaire, diary, emergency department), length of follow-up period, number of patients, mean age of patients, female/male-ratio, monthly frequency of headache days, comorbidities, medication and outcome variables.

The outcome variable was categorized according to the biological rhythm. Following periods were reported: circadian (C), weekly (W) and seasonal (S) distribution of migraine attacks. The three distinct predefined study types were: diary studies, questionnaire studies (relying on patients’ retrospective view of their distribution of attacks) and emergency department studies (analyzing the admittance pattern of patients with migraine diagnosis).

Predefined intervals were applied for studies reporting circadian and seasonal variations of migraine attacks to systemize the data analysis. The 24-hours of the day were categorized into 6-hour intervals: night (00:00 am–06:00 am), morning (06:00 am–12:00 pm), afternoon (12:00 pm–06:00 pm) and evening (06:00 pm–12:00 am). The seasonal distribution of attacks was categorized according to the Northern Hemisphere seasonal standards as all studies were conducted north of the Equator: winter (December–February), spring (March–May), summer (June–August) and fall (September–November).

## Results

**Fig. 1:**
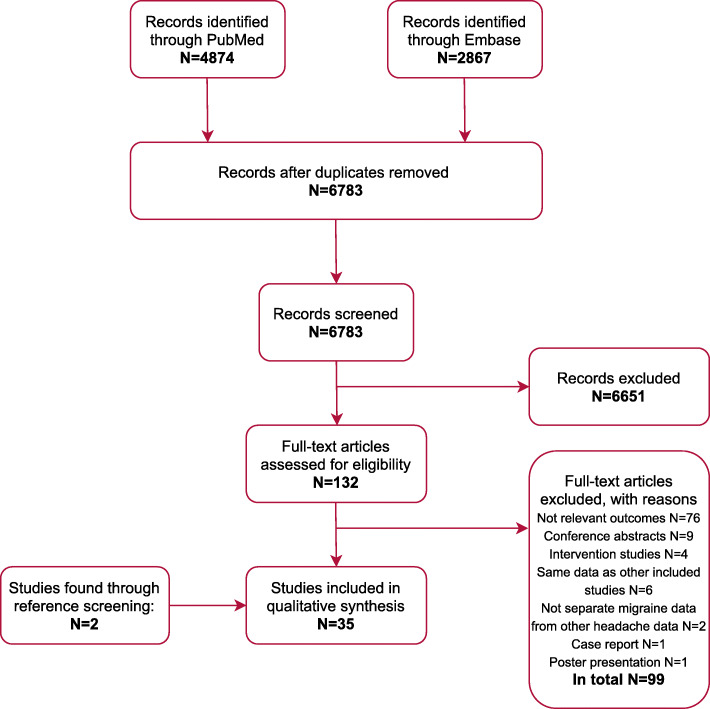
Flow diagram of the review process

The database search resulted in 4874 articles identified through PubMed and 2867 identified through Embase (Fig. 1). Duplicates were removed and a total of 6783 unique articles were retrieved for title and abstract screening yielding 132 articles identified for full-text screening, resulting in 33 eligible articles. Additional two eligible articles were found through reference screening of the 132 full-text screened articles. In total, 35 articles met the eligibility criteria and were included for qualitative synthesis. Distribution of study type among the included articles was: 18 diary studies, 12 questionnaire studies and five emergency department studies.

### Study and patient characteristics

Studies mainly included more migraine patients without aura (MO) than with aura (MA) [5, 16–24]. Six studies included mostly MA patients [3, 18, 25–28]. Furthermore, 12 studies only reported data from patients with episodic migraine (EM) [3, 17, 19, 22, 23, 25–31], while three studies reported data from both chronic migraine (CM) and EM [5, 16, 32]. One study included more patients with CM than EM [16]. All studies were conducted in the Northern Hemisphere [20, 22, 23, 29, 30, 32–38]. Two studies included more males than females [17, 24]. Female/male-ratio was not reported in five studies [35, 39–42]. Mean age was mostly between 30 and 50 years of age (based on 21 out of 24 studies reporting mean age), while three studies investigated a pediatric sample (4–18 years of age) [17, 24, 36] (Table 2).

**Table 2:** Study and patient characteristics

Study	Location	Study type*	Follow-up period (weeks)	Patients (n)	Age (mean $\pm$SD years)	Female/-male ratio	Monthly headache days	Comorbidities (n)	Medication use (n)	Period (C, S, W)
Alstadhaug et al. 2005 [3]	Norway	Questionnaire, retrospective	NA	MA (98) MO (71)	MA: 34.1$\pm$7.2 MO: 32.5 $\pm$8.3	1:0	EM	PCOS (1), depression (2), hypothyroidism (6), asthma and/or allergy (16), hypertension, psoriasis, fibromyalgia and vitiligo	OC (33)	C, S
Alstadhaug et al. 2007 [25]	Norway	Diary, prospective	52	MA (50) MO (34)	35.6 $\pm$6.8	1:0	EM	No concomitant serious disorder	NR	W
Alstadhaug et al. 2007 [26]	Norway	Diary, prospective	52	MA (50) MO (34)	35.6$\pm$6.8	1:0	EM	No concomitant serious disorder	OC (10), beta-blockers (7)	C
Alstadhaug et al. 2007 [27]	Norway	Diary, prospective	52	MA (32) MO (26)	36.9$\pm$6.0	1:0	EM	No concomitant serious disorder	NR	S
Bekkelund et al. 2017 [16]	Norway	Questionnaire, retrospective	NA	MA (106) MO (196)	35.5$\pm$12.6	3.6:1	Monthly migraine days: 0–7 (69), 8–14 (66), ≥15 (166)	Insomnia (95), hypertension (21), chronic neck/shoulder pain (149)	Preventive medication (49), triptans (150), over-the-counter painkillers (267), prescribed painkillers (85)	S
Brewerton et al. 1990 [35]	USA	ED, retrospective	1044	MX (214)	NR	NR	NR	NR	NR	S
Bruni et al. 2004 [17]	Italy	Diary, prospective	2	MO (18)	9.8$\pm$1.2	0.8:1	EM	No concomitant serious disorder	NR	C
Caperell et al. 2014 [36]	USA	ED, retrospective	130	MX (876)	Males: 12.9$\pm$3.1 Females: 13.9$\pm$3.0	1.8:1	NR	NR	NR	S
Cugini et al. 1990 [47]	Italy	Diary, prospective	52	MX (30)	Range: 17–37	1:1	NR	NR	NR	S, W
de Tommaso et al. 2018 [5]	Italy	Diary, prospective	12	MO (538) MO + MA (52) CM (196)	MO: 37.4$\pm$12.5 MO + MA: 34.6$\pm$13.5 CM: 42.5$\pm$14.7	4.0:1	MO (mean$\pm$SD): 7.6$\pm$6.4 CM (mean$\pm$SD): 23.4$\pm$14.8 MO + MA (mean$\pm$SD): 7.7$\pm$6.9	Excluded patients with general medical and/or other neurological or psychiatric diseases	No CNS-active drugs or preventive migraine medication	C
Drescher et al. 2019 [31]	Austria, Germany and Switzerland	Diary, prospective	≥ 13	MX (1085)	43.0$\pm$12.6	4.1:1	EM	NR	NR	W
Gomersall et al. 1973 [18]	Scotland and England	Diary, prospective	26–52	MA (56) MO (30) HM (1)	Range: 11–70	3.7:1	NR	NR	NR	S, W
Gori et al. 2005 [39]	Italy	Diary, prospective	12	MX (100)	38.6$\pm$10.4	NR	NR	NR	NR	C
Hoffmann et al. 2010 [19]	Germany	Diary, prospective	52	MA (4) MO (16)	Range: 18–65	3:1	EM	No other headaches	Preventive medication	C, S, W
Hoffmann et al. 2014 [40]	Germany	Diary, prospective	52	MX (100)	Range: 18–65	NR	NR	NR	Preventive medication	C, S, W
Kelman 2006 [32]	USA	Questionnaire, retrospective	NA	MO and MA** (1283)	37.7$\pm$12 Range: 13-80.5	5.4:1	EM CM	No headaches related to trauma/injury or complicated neurological problems	Preventive medication (269)	C
Kimoto et al. 2011 [20]	Japan	Diary, prospective	52	MA (9) MO (19)	NR	13:2	NR	Hyperlipidemia (3), hypertension (2)	NR	S
Knezevic-Pogancev 2006 [43]	Serbia and Montenegro	Questionnaire, retrospective	NA	MX (2644)	Range: 3–17	1.3:1	NR	NR	NR	C
Lilleng et al. 2009 [21]	Norway	Questionnaire, retrospective	NA	MA (28) MO (60)	40.4$\pm$11.2	1.8:1	NR	No concomitant neurological disorders	NR	S
Marrelli et al. 1988 [41]	Italy	Questionnaire, retrospective	NA	MX and TTH*** (495)	35.8$\pm$12.9	NR	NR	No cluster headache or symptomatic headaches	NR	S
Morrison 1990 [28]	Sweden	Diary, prospective	6	MA (18) MO (17)	41.1	1:0	Migraine attacks/6 weeks: 3.5	NR	Preventive medication (17), antidepressant (3), combined preparation with ergotamine (6), combined preparation without ergotamine (6)	W
Osterman et al. 1981 [45]	Sweden	Diary, prospective	4	MX (53)	40	3.4:1	NR	NR	No preventive medication	W
Park et al. 2017 [22]	South Korea	Diary, prospective	13	MA (1) MO (81)	37.4$\pm 8.3$	5.3:1	6.5$\pm$5.3 (mean$\pm$SD)	No headaches attributed to secondary causes	Preventive treatment (36)	C
Robbins 1994 [37]	USA	Questionnaire, retrospective	NA	MX (494)	Range: 18–60	3.9:1	NR	NR	NR	S
Salvesen et al. 2000 [52]	Norway	Questionnaire, retrospective	NA	MX (289)	40.2 Range: 10–89	3.7:1	NR	NR	NR	S
Shin et al. 2015 [23]	South Korea	Questionnaire, retrospective	NA	MO (769)	48.2 $\pm$12.8	4.4:1	EM	NR	NR	C
Soriani et al. 2006 [24]	Italy	Diary, prospective	52	MO (115)	Males: 9.99$\pm$2.6 Females: 10.2$\pm$2.6 Range: 5–18	0.9:1	NR	NR	No preventive medication that could have modified the rhythm of symptoms	C, S
Szyszkowicz et al. 2009 [38]	Canada	ED, retrospective	NR	MX (64,839)	NR	3.5:1	NR	NR	NR	S
Timothy et al. 2011 [34]	Nigeria	Questionnaire, retrospective	NA	MX (100)	Females: 32.5$\pm$9.9 Males: 31.8$\pm$10.1	2.6:1	NR	No pregnancy or clinical evidence of an organic disease known to cause migraine	Sumatriptan, dihydroergotamine + caffeine and preventive medication	S
Van Oosterhout et al. 2017 [44]	The Netherlands	Questionnaire, retrospective	NA	MX (2389)	45.1$\pm$11.7 Range: 18–74	6.0:1	NR	Lifetime depression (970)	NR	C
Vgontzas et al. 2020 [29]	USA	Diary, prospective	6	MX (98)	35$\pm$12	7.2:1	5.0$\pm$3.6 (mean$\pm$SD)	NR	Preventive medication (26)	C
Villeneuve et al. 2006 [33]	Canada	ED, retrospective	417	MX (4039)	NR	2.9:1	NR	NR	NR	S
Wilkinson et al. 1979 [42]	England	Questionnaire, retrospective	NA	MX (310) Control group MX (100)	NR	NR	NR	NR	NR	C, S, W
Yang et al. 2015 [30]	Taiwan	Diary, prospective	≥ 28	MX (63) Probable migraine (3)****	43.3$\pm$12.9	3.1:1	6.3$\pm$6.2 (mean$\pm$SD)	Hypertension (2), diabetes (6)	Propranolol (40), anticonvulsants (20), flunarizine (13), antidepressant (12)	S
Yilmaz et al. 2015 [46]	Turkey	ED, retrospective	52	MX (3491)	36$\pm 11$	2.6:1	NR	No other diseases causing headache	NR	S, W

Seasonal (S), Circadian (C), Weekly (W), number of patients (n), Not applicable (NA), Episodic migraine (EM), Polycystic ovary syndrome (PCOS), Oral contraceptives (OC), Migraine without aura (MO), Migraine with aura (MA), Migraine, not specified as migraine with or without aura (MX), Not reported (NR), Emergency department (ED), Chronic migraine (CM), Hemiplegic migraine (HM), Tension-type headache (TTH), Standard deviation (SD)

* Method used to collect data: Questionnaire, diary, or emergency department (ED) and retrospective or prospective

**Patients diagnosed with probable migraine are included in the total sample (*n* = 1283); however, outcome is similar to patients diagnosed with MO and MA

***Authors do not report how many participants were included in each group; however, outcomes are reported separately for migraine and tension-type headache

****Outcomes include data from probable migraine patients

### Circadian distribution of migraine attacks

**Fig. 2:**
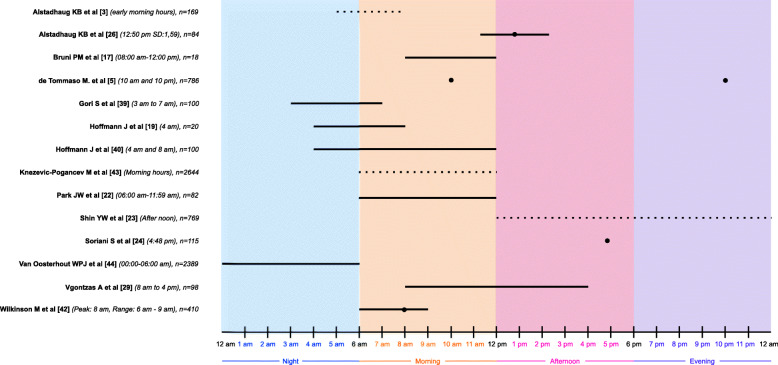
Circadian (24-hour) distribution of migraine attack onset peak interval for the included studies (*n* = 15). One study reported migraine attack onset at any time of the day [32] and thus was not included in the figure. Horizontal black lines indicate the reported time range where most migraine attacks began as reported by the individual studies. Black dot was applied if studies reported a specific peak time. Broken lines were applied for three studies where specific time clocks were unreported [3, 23, 43]. Thus, reporting of early morning hours was estimated to 5 am–8 am [3], morning hours was estimated to 6 am–12 pm [43] and after noon was estimated to 12 pm–12 am [23]. n: total sample size

Fifteen studies investigated the circadian distribution of migraine onset time. Eleven of these studies reported peak time to onset of migraine attacks involving the morning hours (6 am–12 pm) (Fig. 2) [3, 5, 17, 19, 22, 26, 29, 39, 40, 42, 43]. The range of this reported peak overlapped with the nighttime/early morning time interval (3 am–5 am) in four studies [3, 19, 39, 40] and with the afternoon time interval (12 pm–6 pm) in two studies [26, 29].

Three studies did not report peak time to onset of migraine attacks involving the morning hours (6 am – 12 pm). Hereof, one study reported peak at night time (12 am – 6 am) [44], one study reported peak after noon (12 pm – 12 am) [23], while one study calculated peak of attack onset time to 4.48 pm [24]. One study reported migraine attack onset to be at any time of the day [32].

### Weekly and seasonal distribution of migraine attacks

**Fig. 3:**
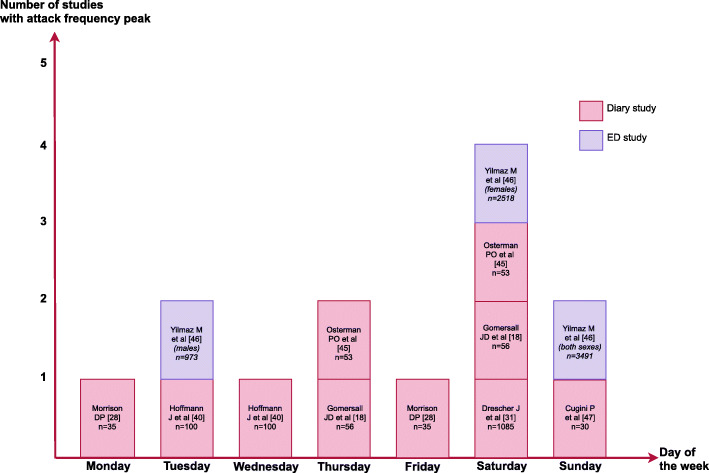
Weekly distribution of migraine attacks. The number of studies reporting migraine attack frequency peak on each day of the week. Three studies found that attacks were equally distributed throughout the week [19, 25, 42]. One study found that there were fewer migraine attacks on Sundays compared to other days of the week [25]. n: total sample size

**Fig. 4:**
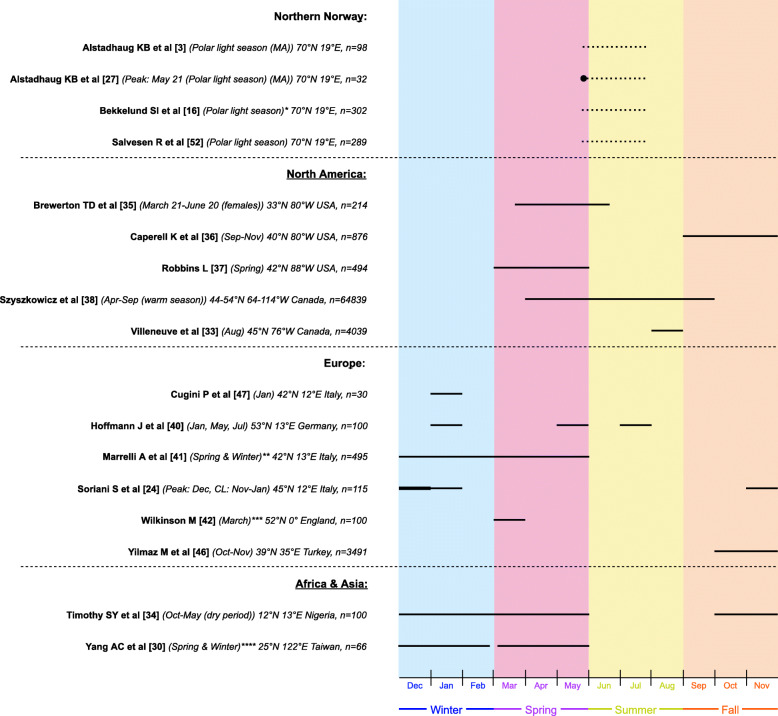
Seasonal distribution of migraine attacks. Horizontal black lines indicate the reported time of year for peak in migraine attack frequency. Broken lines represent estimated dates based on definition of the polar light season in Tromsø (May 21st –July 21st ) [27] as exact dates were not specified by authors. Four studies on the seasonal distribution reported no peak of migraine attack frequency throughout the year [18–21]. Black dot indicates peak date. n: total sample size, N: north (latitude), E: east (longitude), W: west (longitude), MA: migraine with aura. MO: migraine without aura. CL: confidence level. *Data only obtained by those who answered yes to experiencing a seasonal variation of migraine attacks. **Results estimated from figure. Increased headache frequency in spring is more prevalent for MA compared to MO patients. *** This study followed patients for two consecutive years. In the first year, there was no pattern in regard to distribution of migraine attack frequency. In the subsequent year, there was however a monthly variation of attack frequency with a peak in March. **** Temperature sensitive patients showed migraine attack frequency peak in winter and non-temperature sensitive patients had more migraine attacks during spring

Ten studies reported data regarding the weekly distribution of attacks, which is visualized in Fig. 3. Four of the studies reported Saturday as the day of the week where patients experienced more migraine [18, 31, 45, 46]. Twenty-one studies had outcome measures related to seasonal or yearly distribution of migraine attacks. This is visualized in Fig. 4, where studies are grouped according to geographical location. Four out of five studies, conducted in Northern Norway, showed peak of migraine attack frequency during polar light season (May 21st to July 21st [27]), while four out of six studies, in other European countries, most frequently reported peak time interval of migraine attacks during winter [24, 40, 41, 47]. North American studies (*n* = 5) reported peak period of migraine attacks mainly during spring and summer (*n* = 3) [35, 37, 38]. A Nigerian study reported peak of migraine attacks during the dry period (October–May) [34], while one Taiwanese study demonstrated peak during spring and winter [30]. Four studies reported no peak of migraine attack frequency throughout the year [18–21].

## Discussion

This systematic review revealed the association between circadian, weekly and seasonal rhythms and migraine attack periodicity. The majority of studies reported morning hours as being the time of day where most migraine attacks began. Only two studies reported their entire peak of attack onset interval being between 12 pm and 12 am [23, 24]. However, this could be explained by methodological differences such as age differences [24] and study type [23]. The migraine attack onset in morning hours is further confirmed by drug intervention studies [48, 49]. These findings point towards a possible role of the circadian clock in migraine.

This review further revealed a pattern of weekly distribution of migraine attacks. It has been shown that migraine attack frequency rises towards the end of the week peaking on Saturdays [18, 31, 45, 46]. Three [19, 25, 42] out of the ten studies included in the weekly attack distribution analysis could not demonstrate a weekly periodicity phenomenon and reported that migraine attacks were distributed equally throughout the week. It is unlikely that an endogenous infradian rhythm is responsible for migraine attacks being more prevalent on Saturdays than other weekdays. Instead, environmental factors are a more plausible explanation as the transition from weekdays to weekend often is accompanied by life-style changes such as a reduction of perceived stress and changes in alcohol and caffeine consumption [50, 51]. Interestingly, one study found that only patients with a job, and not unemployed patients, experienced fewer migraine attacks on Sundays, indicating that work and work-related stress could influence the weekly pattern of migraine [25].

Seasonal variation of migraine attacks was less clear. Most studies reported that migraine attack frequency was raised during winter and spring rather than summer and fall (Fig. 4). In northern Norway, patients had more migraine attacks during the polar light season (May 21st –July 21st [27]) [3, 16, 27, 52]. Interestingly, two out of four studies only reported this periodicity phenomenon for MA patients and not for the MO patients [3, 27]. One Norwegian study reported no seasonal peak [21]. Given that studies have been conducted at different locations around the world, it is difficult to point at one certain time of the year being worse than another for migraine patients. However, the Norwegian studies suggested that there was a circannual or seasonal periodicity of migraine when comparing studies conducted in the same area with the same climate and endogenous clock settings [3, 16, 27, 52].

### Limitations

The present systematic review revealed limitations of studies investigating a circadian, weekly, and seasonal biological rhythm. Therefore, caution is needed in interpreting the results of these studies. First, the studies were of three distinct study types (diary, emergency department and questionnaire studies) and thus difficult to compare. Headache diary studies may be preferable due to the prospective data collection. Self-reported questionnaire studies are subject to confirmation and recall bias since they rely on patients’ retrospective view of their migraine attack distribution. A recent study showed that patients tend to underestimate their headache frequency using retrospective questionnaire compared to headache diaries [53]. Periodicity of migraine attacks in the emergency department studies might be influenced by various factors. Patients may seek the emergency department due to a long-lasting migraine attack or status migrainosus, and exact time of onset of migraine attack is not routinely recorded. Due to the potential methodological reservations, we marked the emergency department studies separately in this review.

Second, the sample size of the included studies varied widely between and within study types which challenge interpretation of data. Nevertheless, the sample size was relatively larger of studies supporting the circadian pattern of morning hours as the most common time to onset of attacks and Sundays as the potential weekday with peak attack frequency.

Third, the reviewed studies were conducted at different geographical locations and therefore only few studies were comparable regarding the ratio of light to dark hours of the day throughout the year. Melatonin production is the internal representative of the external photoperiod and hereby functions as a chronobiotic hormonal signal controlling both the circadian and circannual rhythms [54]. Thus, only studies with close proximity of geographical location should be compared regarding seasonal variation of migraine to exclude limitations due to different endogenous clock settings.

Fourth, several of the reviewed studies reported other primary outcomes such as the association of migraine to weather [18–20, 30, 33, 34, 40–42, 46] and sleep [17, 29]. Thus, these studies were not designed to mainly consider the periodicity of migraine attacks. Weather and altered sleep rhythm are both considered triggers of migraine and may potentially influence the findings [55].

Fifth, there was lack of information on the clinical characteristics of migraine (e.g., migraine phenotype, attack frequency) in several studies. Periodicity of migraine attacks might differ between patients with and without aura as shown in the reviewed studies [3, 27]. Many studies did not account for whether the attacks experienced by patients were phenotypically migraine attacks. Moreover, it is unclear how many migraine days were reported according to the headache classification, since several studies merely applied the terminology headache days per month. Attack frequency is an important aspect to consider since it would be difficult to estimate a clear pattern of periodicity in CM patients [5].

Finally, several studies failed to provide information on medication use and comorbidities. Certain drugs and diseases can influence the circadian clock system [56, 57] and consequently the periodicity of migraine. Patients using beta-blockers as preventive treatment or oral contraceptives (common treatment of menstrual migraine) had onset of migraine later in the day (4 pm and 3 pm) compared to non-drug users [26]. Furthermore, depression, a common co-morbidity to migraine [58], has been linked to a disruption of the circadian clock system and therefore might have influenced the data [59].

### Lessons learned and future directions

Future research on chronobiology of migraine, including circadian and circannual periodicity, should take into consideration the limitations presented in this review. Studies with refined design aimed to specifically investigate the periodicity of migraine attacks are needed to provide further information on the chronobiology of migraine. As such, prospective diary studies are preferred for data collection following a large sample of well-characterized episodic migraine patients (without preventive treatment). By following the patients for at least one year, we would further be able to establish whether weekly and/or seasonal periodicity is a phenomenon of migraine. Investigations of MA patients separately, and comparing them to MO patients, could reveal if MA patients are more susceptible to seasonal changes. Sex should also be considered in future studies and assessed separately due to the possible influence of menstruation on the periodicity of migraine attacks [60].

Hypothalamus and the pineal gland (hereof melatonin) may be involved in the chronobiology of migraine, since these are the brain structures responsible for circadian and circannual rhythms [61, 62]. Two functional magnetic resonance imaging studies scanned episodic MO patients continuously every morning for 30 days to investigate the hypothalamic activation in migraine [63, 64]. One of the studies reported that hypothalamus was activated up to 48 h before migraine onset [63], while the case study reported increased coupling of hypothalamus to the spinal trigeminal nuclei, 24 h before attack onset, and to the dorsal rostral pons during attacks [64]. This points towards a potential role of the hypothalamus in attack initiation [65]. However, since the time to onset of migraine was not reported in the two neuroimaging studies it is unclear whether the observed hypothalamic activation plays a role in the circadian periodicity. We suggest looking further into a possible role of the hypothalamus to investigate potential underlying mechanisms.

The pineal gland produces melatonin, commonly known as the sleep hormone, which is regulated by the suprachiasmatic nucleus of the hypothalamus, and thus involved in modulating the internal sleep-wake cycle and endogenous biologic timekeeping [66, 67]. The regulation of circadian rhythms is complex and multifactorial [68]. Melatonin has been shown to play a role in regulating the levels of GABA, nitric oxide and CGRP as well as modulating trigeminal activation and neuroinflammation, which are factors involved in the migraine pathophysiology [54]. The question is whether a dysfunction of the retino-hypothalamic-pineal system could influence the circadian timing of migraine onset. A recent systematic review and meta-analysis reported lower levels of nocturnal serum melatonin and urine 6-sulphatoxymelatonin (melatonin metabolite discarded by the urine) in migraine patients compared to healthy controls and that melatonin may be useful as preventive treatment [69].

Interestingly, migraine patients may be less prone to a normal circadian chronotype and more sensitive to changes in their circadian rhythm compared to healthy controls [44]. In support, patients commonly report sleep disturbances as a trigger of migraine [55] and one may suspect that poor sleep quality promote the onset of migraine in the morning hours [13]. However, a recent actigraphy study, investigating patients for six consecutive weeks, reported no association between sleep disturbances and migraine the following day [70]. Further research is needed to establish whether sleep disturbance, low melatonin levels or other regulators of sleep and the circadian clock system, play a role in the periodicity of migraine attacks.

Another question is how periodicity can explain the varying attack frequency within and between patients. Attacks can be triggered by glyceryl trinitrate infusion with the same incidence and severity between patients with rare (≤ 4/year) and frequent (≥ 12/year) migraine attacks [71]. Thus, it is likely that other factors may also be at play.

Migraine patients generally report discomfort to light outside attacks as well [72]. Exposure to light (e.g. amount and intensity) may change across seasons of the year and thus play a role in the migraine periodicity phenomenon. Of note, experimental exposure to photic stimulation was not able to induce migraine attacks in patients reporting light as a common migraine trigger [73]. Nevertheless, abnormal modulation of the complex retino-thalamocortical pathway has been suggested to be involved in photophobia of migraine, which needs further research [74].

## Conclusions

Migraine attacks seem to begin in the morning hours pointing towards a possible role of the circadian clock in migraine. Seasonal and weekly distributions of migraine attacks are less clear, possibly due to heterogeneity within the existing literature. Future studies are needed to further investigate and expand our understanding of the role of chronobiology in the paroxysmic nature of migraine making this an interesting topic for future migraine research.

## Data Availability

The data used in the present review are available from the corresponding author upon reasonable request.

## Abbreviations

C: Circadian
W: Weekly
S: Seasonal
N: Number of patients
NA: Not applicable
EM: Episodic migraine
PCOS: Poly Cystic Ovary Syndrome
OC: Oral Contraceptives
MO: Migraine without aura
MA: Migraine with aura
MX: Migraine, not specified as migraine with or without aura
NR: Not reported
ED: Emergency Department
CM: Chronic Migraine
HM: Hemiplegic Migraine
TTH: Tension Type Headache
SD: Standard deviation
CL: Confidence-level
